# Wildfire policy and management in England: an evolving response from Fire and Rescue Services, forestry and cross-sector groups

**DOI:** 10.1098/rstb.2015.0341

**Published:** 2016-06-05

**Authors:** Rob Gazzard, Julia McMorrow, Jonathan Aylen

**Affiliations:** 1 Forestry Commission England, Forest Services, Bucks Horn Oak, Farnham, Hampshire GU10 4LS, UK; 2 School of Environment, Education and Development, University of Manchester, Oxford Road, Manchester M13 9PL, UK; 3 Manchester Institute of Innovation Research, University of Manchester, Oxford Road, Manchester M13 9PL, UK

**Keywords:** wildfire risk, fire and rescue service, emergency planning, forestry, adaptation, rural–urban interface

## Abstract

Severe wildfires are an intermittent problem in England. The paper presents the first analysis of wildfire policy, showing its halting evolution over two decades. First efforts to coordinate wildfire management came from local fire operation groups, where stakeholders such as fire services, land owners and amenity groups shared knowledge and equipment to tackle the problem. A variety of structures and informal management solutions emerged in response to local needs. Knowledge of wildfire accumulated within regional and national wildfire forums and academic networks. Only later did the need for central emergency planning and the response to climate change produce a national policy response. Fire statistics have allowed wildfires to be spatially evidenced on a national scale only since 2009. National awareness of wildfire was spurred by the 2011 fire season, and the high-impact Swinley Forest fire, which threatened critical infrastructure and communities within 50 miles of London. Severe wildfire was included in the National Risk Register for the first time in 2013. Cross-sector approaches to wildfire proved difficult as government responsibility is fragmented along the hazard chain. Stakeholders such as the Forestry Commission pioneered good practice in adaptive land management to build fire resilience into UK forests. The grass-roots evolution of participatory solutions has also been a key enabling process. A coordinated policy is now needed to identify best practice and to promote understanding of the role of fire in the ecosystem.

This article is part of a themed issue ‘The interaction of fire and mankind’.

## 1. Introduction

### (a) Wildfire policy as an evolutionary process

The UK is vulnerable to wildfires. Their intermittent frequency, poorly documented extent and impact, and the remote location of the largest fires has meant that this semi-natural hazard has been overlooked by policy makers and many local responders until recently. ‘Severe wildfire’ was not recognized as a national risk in the UK until 2013 [1], following the 2011 fire season and the Swinley Forest fire, a relatively small but high-impact fire at the rural–urban interface in South East England [2]. Yet wildfire has been experienced for many millennia [3–5] and climate change is expected to exacerbate the risk [6,7].

Here we review the main challenges facing wildfire management in the UK, but primarily for England.^1^ We show how management structures and policies affecting wildfire have formed in an evolutionary way over the past two decades. Fire and Rescue Services (FRS) have a statutory duty to extinguish wildfires. In the absence of central policy guidance, local community-based solutions to wildfire problems began to emerge during the 1990s. Since then, wildfire policy has exhibited an evolutionary process, with a variety of participatory solutions originating at multiple levels and diffusing in response to need as much as to legislation. Evolution here implies two things: groping for policy solutions in a cumulative and path-dependent way; and separately, the system creates change and emerging structures from variety in behaviour as local groups each develop their own solutions [8].

To begin with, local and regional fire groups developed. Land managers, environmental groups, water authorities and other stakeholders took ownership of the wildfire problem in partnership with local FRS. These self-assembling, informal partnerships developed skills in wildfire management and improved emergency response on the ground. In the process, they raised government awareness of wildfire. National forums were set up in response to crisis events of 2003 and 2006. The England and Wales Wildfire Forum, the Scottish Wildfire Forum and the Chief Fire Officers Association Wildfire Group helped spread good practice through their networks. Policies that impact on wildfire developed separately in each sector. For instance, the Forestry Commission pioneered good practice in adaptive land management to build fire resilience into UK forests.

At the national level, policy responded slowly and fitfully to the complexity of the wildfire problem. The emergency planning and climate change agendas were catalysts for wildfire awareness and the emergence of cross-sector working at national level. Elements of a national policy towards wildfire emerged in 2004 with a programme of contingency planning against risks and natural hazards. By 2010, most Community Risk Registers included ‘forest or moorland fire’. National awareness of wildfire was spurred by the 2011 fire season and risk assessments for the 2012 London Olympics. Severe wildfire was included in the National Risk Register for the first time in 2013. The need for cross-sector collaboration on wildfire was highlighted by the Climate Change Risk Assessment in 2012.

### (b) The context of wildfire in the UK

Only one-tenth (2.6 million ha) of the UK is urbanized. For the most part it is countryside, with agriculture (crops, grazing, grassland, etc.) comprising almost 65% (over 15.3 million ha), followed by forestry at 13% (over 3 million ha) [9]. Fires often occur at the ‘rural–urban interface’, where countryside meets settlements. There are no true wilderness landscapes, so the term ‘wildland–urban interface’ used elsewhere in the world seems inappropriate.

**Figure 1.**
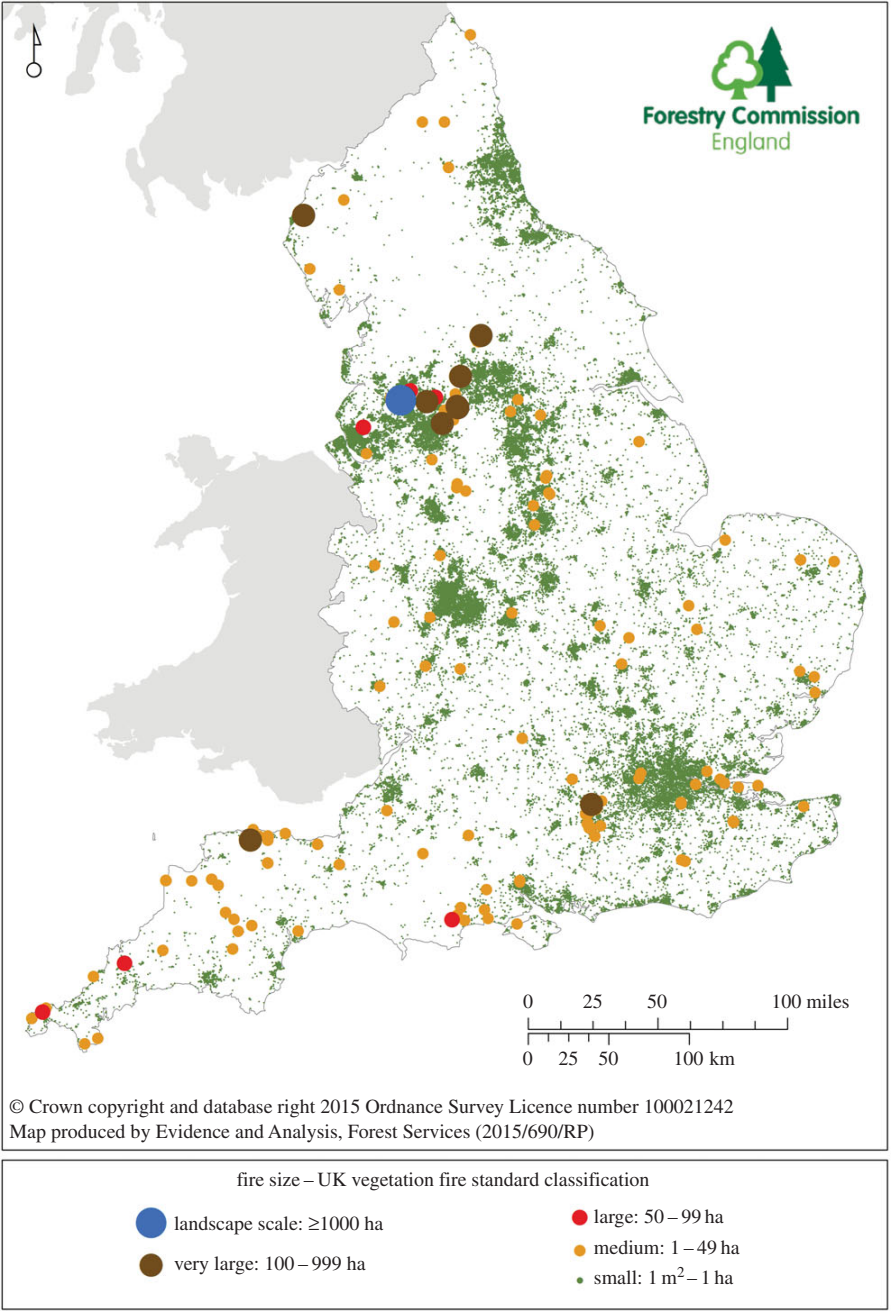
Distribution of vegetation fires recorded in the Incident Recording System for England by size class, financial year: 1 April 2011–31 March 2012. (i) Symbols are not directly proportional to fire size; (ii) size classes are as defined in the UK Vegetation Fire Standard; (iii) Incident Recording System data are courtesy of Department for Communities and Local Government.

A wildfire in the UK context is ‘any uncontrolled vegetation fire which requires a decision, or action, regarding suppression’ [10, para. 3.3, p. 10]. Analysis by the Forestry Commission^2^ of FRS’ Incident Recording System data for Great Britain (GB—UK excluding Northern Ireland) [11] shows that 210 000 such incidents were recorded by FRS annually in financial years 2009/2010 and 2012/2013, with 65 000 alone in 2010/2011, burning an estimated area of over 71 000 ha. Most were on the fringe of urban areas, in the rural–urban interface (figure 1). Given the liberal definition of wildfire, 49% were under 5 m^2^ and accounted for under 0.1% of the estimated burned area. Of these, 83% were recorded as Incident Recording System property types, ‘tree scrub’, ‘grassland, pasture, grazing, etc.’, ‘scrub’ or ‘urban types’^3^ [12]. In counties like Dorset, however, 25% of fires are small but intense lowland heath fires, with potentially high social costs when they are located near densely populated areas and critical national infrastructure. Indeed, 99% of GB fires are under 1 ha not only because of the relatively small, discontinuous patchwork of fuels, but because of early FRS suppression to avoid the potentially high social costs from, for example, traffic disruption and the health effects of smoke.

Such fires attract political attention when major assets are threatened, as in the widely reported Swinley Forest fire in South East England in May 2011, just 50 miles west of London [2]. It was one of the most resource-intensive fire incidents in Berkshire since World War II.^4^ Media coverage was intense, compared with the much larger, concurrent but relatively remote moorland fires.^5^ Smoke was visible from a local MP's home and the issue was raised in the UK Parliament [13].

Forestry Commission analysis of Incident Recording System data^6^ shows that while open habitats represented only a small fraction of wildfires, they accounted for nearly 80% of the estimated area burnt in GB. In particular, mountain, moorland and bog fires accounted for 40% of area burnt, because horizontal continuity of fuel, topography and the difficulty of suppression allow fires to spread. During financial year 2011/2012, they accounted for 49% or over 18 000 ha.

Moorland wildfires attract limited political interest because they rarely threaten life, key infrastructure and property. However, their environmental costs can be high when they burn into peat, releasing carbon into the atmosphere and triggering peat erosion. Such peat fires silt up reservoirs, discolour drinking water, may release heavy metals from past pollution, and damage wildlife habitats and landscape quality [14,15]. Ecological restoration of peatland burn scars in the Peak District National Park in North West England has cost over £12 million [16]. Suppression costs of moorland fires are high due to problems of access and water supply—up to £1 million for a large peat moorland fire [17]. Annual average costs to FRS of vehicle response to all vegetation fires in GB were estimated at up to £55 million.^7^

At the same time, it is important to recognize that vegetation fire can bring benefits, depending for instance on the severity and frequency of a burn and the ecosystem service prioritized [18]. Benefits include habitat diversity, control of some insects and diseases, recycling nutrients and encouraging fire-dependent species [19]. Fire is a traditional management tool, used to maintain grassland for grazing and heather moorland for game bird shooting. Heather moorland is a fire-adapted ecosystem maintained by rotational prescribed burning to give a mosaic of mixed-age heather for grouse (notably *Lagopus lagopus scotica*) [20]. Whether land management burns prevent or cause peatland wildfires is highly controversial [18]. Heather and gorse on lowland heaths found in areas like Dorset in South West England and East Anglia are also fire-adapted ecosystems, where fire assists regeneration, encouraging seed germination and preventing succession to scrubland [21,22].

## 2. Challenges for wildfire management in the UK

Severe wildfire was recently officially recognized as a semi-natural hazard by central government, largely due to the unusual nature of its impacts. This slow recognition can be linked to a range of challenges including the sporadic timing of major events, historically weak reporting requirements and the fragmented, silo structure of management, which inhibits a cross-sector view for national policy.

### (a) Episodic timing

Vegetation fires in the UK are most prevalent in spring, when there are dry fine fuels from dead vegetation after winter freezing and drying, and during hot summers [23,24]. Although wildfires occur annually, the most serious incidents are intermittent, being concentrated in just a few dry years, 1976, 2003, 2006 and 2011, for example [25]. This intra-annual variability and the clustering of incidents over short periods, such as a week or fortnight, makes severe claims on FRS resources [26]. Wetter years mean fewer opportunities to gain experience in tackling real incidents. As a result, wildfires move off the political agenda, and it is hard to justify intermittently used specialized equipment and training. There is also little recognition that within the short time frames of politics and emergency planning (4–5 years), a sequence of warmer, wet years may be promoting fuel build-up, which in other parts of the world has led to more intense fires [27].

### (b) Limited evidence base

Limited requirements for reporting of vegetation fire incidents by individual FRS meant that there was no nationally consistent and complete statistical evidence to support national policy decisions on wildfire until 2009, when the Incident Recording System was rolled out [28]. However, the accuracy of the system depends on the competency of the FRS personnel inputting data. The system omits escaped land management burns not reported to FRS. Consistent long-run statistics are still lacking, but now that all vegetation fire incidents attended by FRS are individually reported to central government and are geo-referenced, spatial analysis of Incident Recording System data is allowing a clearer picture of wildfire geography for England, Wales and Scotland^8^ to emerge (e.g. figure 1). Biannual and annual reporting of these fire statistics by central government takes up to six months to collate and check and, despite the potential for mapping, is usually non-spatial [29].

A further challenge is that the Incident Recording System covers a financial year, 1 April to 31 March, which splits the main spring fire season. Another difficulty is agreeing a definition of wildfire that allows the more significant incidents in the System to be differentiated from smaller vegetation fires, while allowing for differences in suppression resources and strategies between fire services. There are now 50 FRS in GB; 46 in England (with potential reorganization), three in Wales and one in Scotland (eight Scottish FRS merged in 2014). The old ‘primary’ and ‘secondary’ fire categories were based on resources deployed or impact on structural property (buildings) and human safety [29, p. 8]. Scotland's wildfire operational guidance has a more flexible definition of large wildfire for recording purposes, using any one of five criteria: involving a geographical area of greater than 1 ha; requiring a committed resource of greater than or equal to four fire appliances; requiring resources to be committed for greater than or equal to 6 h; having a sustained flame length of greater than 1.5 m; or presenting a serious threat to life, environment, property and infrastructure [10, para. 3.4, p. 10].^9^ Flame length is not yet recorded in Incident Recording Systems and not all impacts are captured.

Finally, a fire is recorded as a single point, so further challenges lie in improving spatial accuracy, preferably to the suspected ignition point, and the need to collect fire perimeters (as already done in Dorset and the Peak District National Park). Perimeters enable burned area to be calculated instead of visually estimated. Geospatial analysis could then be used to quantify assets at risk and calculate metrics such as fire recurrence interval or Fire Potential Index [31]. Use of satellite remote sensing as an alternative data source [32] is severely limited by cloud cover, small fire size or duration, and smoke from peat fires [33], although Synthetic Aperture Radar shows promise for detecting burn scars in fire-degraded peat moorland [34].

### (c) No national wildfire agency; wildfire as a regional fire service issue

The UK has no single agency or firefighting force with specific responsibility to manage wildfire. Instead, statutory responsibility rests with individual FRS under the Fire and Rescue Services Act 2004, or equivalent for the devolved administrations. FRS training and equipment focuses on fighting fires in urban buildings, handling emergencies such as chemical spills and rescue from road traffic accidents. Most Services had little knowledge and understanding of rural wildfires 10 years ago. The penetration of wildfire culture into organizations is improving but still depends upon inter-generational championing and knowledge exchange. Wildfire knowledge and practical skills are lost due to staff rotation and retirement, both in FRS and the rural land management community. This limits opportunities to build community and institutional resilience through an iterative cycle of management, social learning and adaptation [35], although we later discuss the progress made by Northumberland FRS [36] and other wildfire champions [37, p. 15].

### (d) Functional fragmentation of the hazard chain

Wildfire has been aptly portrayed as a ‘wicked problem’; one that is hard to define, has multiple causes and poses contradictory or changing requirements [38]. Initial solutions to wicked problems are prone to reveal further problems because of cross-sector implications, so that wicked problems are seldom the responsibility of just one entity. In England, however, the wildfire hazard is officially ‘owned’ by the government Department for Communities and Local Government (DCLG), which has overall responsibility for Fire Authorities and their FRS (table 1), and the built environment. Responsibility for Fire Authorities will transfer to the Home Office from April 2016.

**Table 1.** Structure of wildfire management in England; sectors, key agencies and their contributions to wildfire management.

sector	organization/agency/group	scale	contributions to wildfire management
Contingency planning	— Cabinet Office Civil Contingency Secretariat	national	— National Risk Assessment and public-facing version, National Risk Register. — Severe wildfire H58 added in 2013. Renewed in 2015
	— Department for Communities and Local Government Resilience and Emergency Planning Directorate	national	— Government department with ownership of wildfire hazard under Civil Contingencies Act 2004—due to change to the Home Office in April 2016 — Produce Fire Statistics GB; annual bulletin including statistics on outdoor fires attended by FRS
	— Scientific Advisory Group of Experts	national	— Co-opted advice from experts at times of emergency
	— Local Resilience Forums based within 39 Police Areas in England; cross-sector category 1 and 2 responders (police, fire, ambulance, etc.) and Local Authority emergency planners	regional	— Community Risk Registers which rank likelihood, impact and risk of ‘forest or moorland fire’ and ‘severe wildfire’ within a five-year period relative to other local risks
Fire	Chief Fire Officers Association, Wildfire Group, National Operations Programme Group	national	— Non-statutory groups working to develop best practice in recording and managing wildfire, e.g. Development of National Operations Programme Group wildfire guidance, and introduction of Firewise communities
	49 FRS in England and Wales, managed by regional Fire Authorities, and overseen by the DCLG due to change to the Home Office in April 2016 Single service in Scotland since 2014 Single service in Northern Ireland since 1950s	regional	Statutory — Wildfire suppression — Incident Risk Management Plans — Incident Recording System since 2009, previously Fire Data Report System — Provide agreement on wildfire prevention in Environmental Impact Assessments, Environmental Statements and some Countryside Stewardship options Voluntary — Wildfire Operational Guidance (The Scottish Government 2013) — Wildfire training developed by Northumberland FRS and other pioneering FRS
Environment	DEFRA Wildfire Group, within DEFRA Contingency Planning Team	national	— Advice to Cabinet Office Civil Contingency Secretariat and DCLG's Resilience and Emergency Planning Directorate for National Risk Register — Government and agency environmental and rural experts at times of emergency — Chair the former Best Practice Burning Group which consulted on Heather and Grass Burning Regulations 2007 and associated Code — Countryside Stewardship grants with wildfire prevention requirements for moorland and heathland
	Forestry Commission	national to local	— Champion of UK Forestry Standard, and guidelines linked to planning for wildfire in woodland and forests — Wildfire practice guidance for land managers — Wildfire contingency planning for forestry sector — Highlighting wildfire risk in deforestation Environmental Impact Assessments — Wildfire analysis of FRS Incident Recording System data for Great Britain GB — Impact of wildfire emissions and carbon storage
	Met Office	national	— Met Office Fire Severity Index developed for Natural England to regulate public access to statutory Access Land under the Countryside and Rights of Way Act 2000
	Natural England	national to local	— Management plans for statutory protected areas—recent consideration of wildfire risk management — Member of former DEFRA Best Practice Burning Group, now Uplands Management Group — Countryside and Rights of Way Act 2000; open-access land wildlife restrictions (linked to Met Office Fire Severity Index)
	Wildlife and landscape conservation groups: Royal Society for the Protection of Birds (RSPB), National Trust, Wildlife Trusts, etc.	national to local	— RSPB as a member of former DEFRA Best Practice Burning Group, now Uplands Management Group — National Trust as a member of some local fire operations groups — Wildfire suppression — Land management of sites threatened by present and future wildfire incidents
	Land management community: practitioner associations such as the Moorland Association, the Heather Trust, Game and Wildlife Trust	national to local	— Tacit knowledge and skills in use of prescribed fire for moorland management — Members of former DEFRA Best Practice Burning Group, now Uplands Management Group — Membership of informal local wildfire groups — Key consultees for policy, plans and guidance — Wildfire prevention, suppression and recovery — Land management of sites threatened by present and future wildfire incidents
Under-represented sectors	Development control planning	regional to local	— National Planning Policy Framework (table 2), but relatively little engagement — Dorset and Thames Basin Heaths financial contributions from planning applications to help prevent heathland fires
	Department for Energy and Climate Change	national	— Consultations for Climate Change Risk Assessment and National Adaptation Plan (table 2)
	Insurance industry		— Insure land managers against escaped management burns
Cross-sector	England and Wales Wildfire Forum, Scottish Wildfire Forum	national	— Voluntary advocacy coalitions on wildfire. ‘go-to’ groups advising government Memberships spans all sectors listed above and others including infrastructure (Highways Agency, Network Rail, Ministry of Defence, etc.)
	Local wildfire groups, also known as Fire Operations Groups	regional and local	— Voluntary partnerships of local stakeholders including FRS, local land owners and managers and government agencies. Share equipment and training, develop Fire Plans
	Academic-led initiatives: e.g. — FireBeaters, 2006–2009, Edinburgh University — Fire Interdisciplinary Research in Ecosystem Services: fire and climate change in UK moorlands and heaths (FIRES) seminar series 2007–2009, University of Manchester with Universities of Edinburgh and Leeds, and Moors for the Future partnership. Peak District National Park — Knowledge for Wildfire (KfWf) Knowledge Exchange Fellowship, 2012–2016, University of Manchester	national	— FireBeaters collected data on vegetation fire incidents and carried out research on moorland fire behaviour — FIRES developed a cross-sector and cross-disciplinary community of researchers, practitioners and policymakers who work on prescribed fire and wildfire in the UK Produced FIRES policy brief [39]. Funded jointly by Economic and Social Research Council and Natural Environment Research Council — KfWf knowledge exchange network, events and projects, funded by the Natural Environment Research Council

Wildfire is *de jure* an FRS problem, but *de facto* it is also a land and people management problem that falls within the scope of many agencies and sectors (table 1). Management is functionally fragmented between them, each operating within its own policy silo (table 2). Consequently, policies that affect land management or people can inadvertently affect wildfire risk by changing fuel conditions; for example, by rewilding or withdrawal of agri-support schemes, or by increasing the density of ignition sources and demand for suppression through improved public access to greenspace or new-build housing in the rural–urban interface. Fire Services engage in wildfire prevention such as public education initiatives, but they have no legal jurisdiction over public access or fuel management.

**Table 2.** Legislation, policy and plan implications and opportunities for wildfire in England.

legislation	implications and opportunities for wildfire
Civil Contingency Act (2004)^a^	— National Risk Assessment and National Risk Register to be undertaken by Cabinet Office Civil Contingency Secretariat — Local Resilience Forums to prepare a Community Risk Register — Fire and Rescue Authorities have a duty to ‘assess, plan and advise’ on situations which threaten serious damage to the environment such as wildfires if defined as a significant risk in the Community Risk Register
Fire and Rescue Services Act (2004)^b^	— Required Fire Authorities required to make provision for the purpose of promoting fire safety in their area — Have the core duty to extinguish fires in their areas to protect life and property. This includes wildfire — Must have regard to the National Framework in carrying out their functions
Regulatory Reform Order (Fire Safety) (2005)^c^	— Forestry and agriculture excluded from fire precautions, risk assessment, prevention and other arrangements for preventing wildfire incidents
Forestry Act (1967)	— No link to wildfire within the Act
Countryside and Rights of Way Act (2000)^d^	— Powers to restrict or exclude access for the avoidance of risk of fire or danger to the public (§25 (1, 2, 3 and 4)) — Use of the Fire Severity Index to trigger fire prevention restrictions on Access Land mapped under the Countryside and Rights of Way Act (2000)
Wildlife and Countryside Act (1981)^e^	— Section 28G authorities, such as FRS and government, local authority and agency landowners, have a general duty to take reasonable steps, consistent with the proper exercise of the authority's functions, to further the conservation and enhancement of the flora, fauna or geological or physiographical features by reason of which the location is a Site of Special Scientific Interest
Town and Country Planning Act (1990)^f^	— Defined the need for local authorities to provide structure plans for development as well as development control — Wildfire is defined via the National Planning Policy Framework within structure plans and submitted development proposals
Water Environment (Water Framework Directive) (England and Wales) Regulations 2003^g^	— Impact of wildfire on water quality for human consumption
Climate Change Act 2008^h^	— Established a framework to develop an economically credible emissions reduction path. This requires the government to: undertake an assessment, i.e. Climate Change Risk Assessment, and a plan (National Adaptation Plan) to assess the UK's risks from climate change, prepare a strategy to address them, and encourage critical organizations to do the same — DEFRA is the lead government department for domestic adaptation policy — Wildfire is identified in both the Climate Change Risk Assessment and National Adaptation Programme

policy	implications and opportunities for wildfire
National Fire and Rescue Framework 2012	— Set out the three priorities for Fire and Rescue Authorities that are linked to building wildfire resilience: — identify and assess the full range of foreseeable Fire and Rescue-related risks their areas face (such as wildfire), make provision for prevention and protection activities, and respond to incidents appropriately — work in partnership with their communities (such as the land management sector) and a wide range of partners locally (such as wildfire groups) and nationally (such as wildfire forums) to deliver their service — be accountable to communities (such as land manager and owners) for the service they provide
National Planning Policy Framework 2012^i^	— To take into account climate change and natural hazard effects and to anticipate the impact of incidents (such as wildfires). Also to provide mitigation and adaptation within development proposals and plans

plans and reports	implications and opportunities for wildfire
Keeping the country running: natural hazards and infrastructure 2011^j^	— Identifying and assessing risks and types of natural hazards that could affect national infrastructure. Wildfire is defined in the context of an emergency, as defined in the Civil Contingency Act, as a knock-on effect of natural hazard events, such as reduced rainfall, prolonged periods of warm weather and storms/gales
Climate Change Risk Assessment 2012^k^	— Sets out the main priorities for adaptation in the UK under five key themes identified in the Climate Change Risk Assessment 2012 Evidence Report: Agriculture and Forestry; Business, Industries and Services; Health and Wellbeing; Natural Environment; and Buildings and Infrastructure — Describes the policy context, and action already in place to tackle some of the risks in each area — Wildfire is defined under Biodiversity and Ecosystem Services as a cross-sector risk with various impacts
National Adaptation Programme 2013^l^	— The National Adaptation Programme contains a register of actions, including those relating to wildfire. It also aligns risks identified in the Climate Change Risk Assessment to actions being undertaken or to be undertaken and the time scales according to each theme. These actions are across a broad range of stakeholders, including land managers, Fire and Rescue Services FRS, and organizations such as the Chief Fire Officers Association and the England and Wales Wildfire Forum
Chief Fire Officers Association's (CFOA) Climate Change Adaptation Report 2014^m^	— Identifies wildfire as a high-priority risk across four of five sector themes — Defines the need to address wildfires with specialist vehicles and equipment and partnership working, and proposes options for actions and analysis
Natural England's Climate Change Risk Assessment and Adaptation Plan (2012)^n^	— Defines Natural England's strategic direction to protect biodiversity and increase resilience — Wildfire is defined as a key high-priority risk across a wide range of themes, including how designations are effected, e.g. National Nature Reserves and land management funding
United Kingdom Forestry Standard 2011^o^	— Defines how sustainable forestry management can be achieved to meet international and European agreements as well as meeting national legislation and good-practice requirements, with supporting guidelines — Wildfire is defined as a risk along with criteria to mitigate and adapt

^a^http://www.legislation.gov.uk/ukpga/2004/36/contents

^b^http://www.legislation.gov.uk/ukpga/2004/21/contents

^c^http://www.legislation.gov.uk/uksi/2005/1541/pdfs/uksi_20051541_en.pdf

^d^http://www.metoffice.gov.uk/public/weather/fire-severity-index/#?tab=map&fcTime=1444734000&zoom=5&lon=-4.00&lat=55.74

^e^http://www.legislation.gov.uk/ukpga/1981/69/contents

^f^http://www.legislation.gov.uk/ukpga/1990/8/contents

^g^http://www.legislation.gov.uk/uksi/2003/3242/contents/made

^h^http://www.legislation.gov.uk/ukpga/2008/27/contents

^i^https://www.gov.uk/government/publications/national-planning-policy-framework--2

^j^https://www.gov.uk/government/publications/keeping-the-country-running-natural-hazards-and-infrastructure

^k^https://www.gov.uk/government/publications/uk-climate-change-risk-assessment-government-report

^l^https://www.gov.uk/government/publications/adapting-to-climate-change-national-adaptation-programme

^m^https://www.cfoa.org.uk/download/4971

^n^http://publications.naturalengland.org.uk/publication/216300

^o^http://www.forestry.gov.uk/ukfs

Effectively, responsibility for these two key elements of the prevention phase—public access (ignition sources) and land management (fuel)—rests largely with the Department for Environment, Food and Rural Affairs (DEFRA). The Met Office (a Ministry of Defence agency) produces an online Fire Severity Index which is used to trigger closure of statutory public Access Land in England and Wales under the Countryside and Rights of Way Act at times of exceptional fire risk (table 2) [40]. DEFRA and one of its agencies, Natural England, have responsibility for land management policy through agri-environment subsidies and wildlife conservation. As we will show, the Forestry Commission (another of the DEFRA family) has played a pivotal role in initiating wildfire awareness and planning at the prevention stage within DEFRA. To a smaller degree, the Department for Transport has responsibility for transport infrastructure, and the Department of Energy and Climate Change for adaptation measures and impacts on energy production and infrastructure.

In the preparedness and emergency response phase, responsibility passes currently to DCLG and thus FRS. For severe incidents, however, a multi-agency response is adopted during the response phase, as with other hazards. In the recovery phase, responsibility passes back to DEFRA agencies. Thus, there is a clear disconnect between the holistic approach required and fragmentation of management at different phases of the whole hazard chain.

We will show that community-based cross-sector networks have emerged to redress this disconnect, as have cross-agency working groups; for example, DEFRA's current Uplands Management Group. Central Government's growing recognition that wildfire is not just a fire service issue but also a land management and environmental one is recent, and was catalysed by the climate change agenda and Forestry Commission initiatives.

### (e) Suppression paradigm

National policy is to intervene relatively late in the hazard chain, focusing on suppression, with less attention to the socio-ecological context in which fires start and spread [41]. While suppression may be the appropriate response in a densely populated country like the UK, there are dangers inherent in zero tolerance. In fire-prone countries, removing all fire from an ecosystem in the absence of other measures allows fuel to accumulate, increasing the danger of more intense future fires [42]. Fuel load management through burning, cutting or grazing, and working collaboratively with land managers to improve prevention, preparedness and response to wildfires, may reduce FRS call-outs and help firefighter safety [26, p. 91]. Improving ecological resilience by re-wetting degraded peat moorlands may also reduce incidents [16].

Given DCLG's current ownership of the wildfire hazard, and through DCLG, FRS, it is not surprising that the dominant paradigm is suppression of all vegetation fires attended by FRS. The norm is to view any such vegetation fire as hazard. The strong focus on carbon and peatlands in the UK, for instance by the International Union for the Conservation of Nature's UK Peatland Programme [43], has tended to reinforce the view that all fire on moorlands overlying deep peat is bad, whether wildfire or controlled land management burns [44]. Restrictions on agricultural straw burning in England and Wales after 1993 [45] are another sign that fire is being excluded from rural culture, rather than being seen as part of the process of land management.

In the context of these challenges, we now examine the management structures and policy affecting wildfire, which have developed over the past two decades, focusing particularly on England (tables 1 and 2).

## 3. Local and national management responses to wildfire in England

Policies towards wildfire have gradually emerged over time, driven by the evolving emergency planning and climate change agenda, the advocacy of wildfire champions and by crisis events, notably the Swinley Forest rural–urban interface fire in April/May 2011.

### (a) Community-based cross-sector working

Policy towards wildfire in the UK shows a pattern of evolution, starting with recognition of a problem followed by gradual emergence of solutions. Community-based solutions first appeared at local, regional and then national levels during the 1990's, long before formal awareness and government policy began to deal with the issue. These local wildfire groups and later regional and national forums evolved in response to crisis events. A patchwork of local solutions developed in Scotland, across England and in Wales from this bottom-up process. Knowledge of wildfire management has grown within these informal networks and diffused upwards from local and regional to national.

#### (i) Local and regional fire groups

Local stakeholders have taken ownership of the wildfire problem, forming wildfire groups in collaboration with local FRS and working across conventional institutional boundaries. The Peak District Fire Operations Group was founded in 1997 [46]. It exemplifies innovation distributed across an informal network, with the Peak District National Park Authority acting as initiator and knowledge broker. Members include the six FRS within the Park, local water companies, amenity groups and landowners. It became a model for other local wildfire groups, as has its Scottish equivalent, the South Grampians wildfire group.

Knowledge on the management of wildfires has been co-produced by these self-assembling local groups. Examples include local fire plans with inventories of firefighting equipment, emergency contacts, vehicle rendezvous and access points, and sources of water for firefighting. Neighbouring FRS collaborate, adopting the same size hoses and couplings to overcome problems of interoperability. This knowledge-making has been spontaneous and stakeholder-led [47]. These groups have developed new ways of working—a process of resourceful improvisation known as ‘bricolage’ [48]—pulling in new stakeholders where appropriate. For example, the Peak District Fire Operations Group wildfire suppression training programme included novel topics such as working with helicopters, thereby co-opting pilots as new stakeholders into the wildfire management process. In this way, the local Group was re-negotiated to incorporate new members as understanding of the problem accumulated with cooperation, experience and induced inventive learning [49] to form a high-reliability network [50].

The Peak District Fire Operations Group was copied elsewhere, for example, in South East England, Northumberland and Cumbria [51–53]. As a result, knowledge of wildfire management began to be distributed horizontally around informal networks across the UK, with each group pursuing locally situated agendas [54]. The Peak District developed expertise in rapid fire suppression using helicopters, as their overriding priority was to prevent damage to peat and drinking water supplies. Northumberland developed skills in back burning, borrowed from Catalonia, as a low-cost technique to control fire spread, as they had few firefighting resources in a sparsely populated county. Back burning and other associated ‘indirect’ attack techniques have diffused across the UK to gradually modify firefighting practice elsewhere, notably in Scotland, but also in the later stages of the Swinley fire. In this fashion, a grass-roots response to local problems generated a variety of outcomes. But the uneven spatial coverage of fire groups and the absence of a national integrated approach to wildfire management mean that an institutionally fragmented approach to managing wildfire still persists.

Tensions may exist within cross-sector groups [55]. Fire has long been used as a management tool in UK uplands, but traditional practice does not meet with approval from those concerned with damage to peat, birdlife or water quality. Moorland managers and nature conservation groups have divergent attitudes to controlled burning on upland peat, including whether burning reduces wildfire risk through fuel management, or increases it due to escaped fires and by promoting more fire-prone, less ecologically resilient habitats. Yet these rival groups coalesce on the need to manage uncontrolled wildfire. Preventing severe wildfire is a uniting boundary concept that both can buy into.^10^

The wider outcome has been a pattern of bottom-up innovation characterized by new forms of management, extensive collaboration across organizational boundaries on issues such as fire plans, new items of equipment and specialized training, well outside the national Fire College training framework.

#### (ii) The England and Wales Wildfire Forum

This spread of knowledge developed as regional and national coordinating groups emerged, fostered by key champions. These groups might be variously seen as ‘advocacy groups’, ‘communities of practice’ or ‘networks of practice’ [56]. The Scottish Wildfire Forum was set up in 2004 after severe wildfires in the hot, dry spring of 2003. The model was copied by the English Wildfire Forum in November 2007, spurred by the 2006 wildfire season and following discussions between the Chief Fire Officers Association, the Forestry Commission and Natural England. It was led by Mark Jones, then Deputy Chief Fire Officer in Essex, who had earlier been instrumental in setting up the Scottish Wildfire Forum while at Grampian Fire Service [37, p. 15]. When Northumberland FRS took over in 2010, they proactively expanded membership into the England and Wales Wildfire Forum.^11^ Members now include FRS, DCLG, the Cabinet Office, DEFRA agencies (Forestry Commission England, Natural England), Ministry of Defence (including the Met Office), Natural Resources Wales, Ministry of Defence, infrastructure agencies (e.g. Network Rail, Highways Agency), Local Government Association, National Park Authorities, private land management groups (e.g. Heather Trust, Moorland Association) and researchers, among others.

Both national Forums are therefore cross-sector, multi-agency groups of public, private and third-sector stakeholders established to address wildfire issues but they are non-statutory. In part, they are a response to the spatially uneven coverage of local fire groups and to national fragmentation of responsibility. Their roles include coordination, lobbying for change, serving as centres for knowledge exchange and a point of consultation for government bodies.

#### (iii) Knowledge exchange

University-based knowledge exchange projects have also helped build a cross-disciplinary and cross-sector national wildfire community. Initiatives have included: FireBeaters (2006–2008) [57]; the Fire Interdisciplinary Research on Ecosystem Services^12^ (FIRES) 2007–2009 seminars and resulting influential policy brief [37,39, p. 16], whose recommendations directly influenced the Chief Fire Officers Association Wildfire Group's action plan; collaborative heather burns with Northumberland FRS and five universities [36, p. 35, 58]; and Knowledge for Wildfire, 2012–2016.^13^

### (b) Emergency planning response to wildfire

National policy towards wildfire began to develop as part of a wider appreciation in government after ‘9/11’ that modern society faces malicious and natural risks which require risk assessment and contingency planning. In the process, wildfire was recognized as a semi-natural hazard (the vast majority are started by humans), with unique impacts facing civil society and one exacerbated by climate change.

Official recognition of wildfire as a significant hazard can be traced back to the Civil Contingencies Act 2004, which addressed any emergency that threatened serious damage to human welfare, environment and the security of the UK [59]. It required a National Risk Assessment to be undertaken by the Cabinet Office and other government bodies, and allocated a lead government department to drive the response forward (table 2). While the Act required multi-agency collaboration, the choice of DCLG as lead confirmed the Fire Service-centric approach to wildfire risk management. While recognition on the National Risk Register undoubtedly raised the profile of wildfire widely within and beyond the fire service, it also reinforces the view of vegetation fire as hazard, with no ecological or fuel management benefits.

#### (i) National Risk Register

The severe spring fire season of 2011, and especially the Swinley Forest fire, together with contingency planning for the 2012 London Olympics, brought two changes in government policy towards wildfire. First, in 2013, severe wildfire was added to the National Risk Assessment and its public-facing version, the ‘National Risk Register of Civil Emergencies’ [1], which was reconfirmed in 2015 [60]. DCLG was defined as the lead organization for wildfires. Second, in late 2011, the Cabinet Office Civil Contingencies Secretariat produced a guide to improve the resilience of critical infrastructure and essential services. It recognized wildfire as an emergency in its own right, distinct from other major fire types, and a knock-on consequence of ‘natural hazards’ such as prolonged periods of dry and/or hot weather [61].

#### (ii) Local Resilience Forums and Community Risk Registers

The Civil Contingencies Act, §1, also required Local Resilience Forums to undertake contingency planning. Local Resilience Forums are multi-agency, consisting of Category 1 responders in a Police Area (emergency services, including Fire Authorities^14^), Category 2 responders including Local Authorities, the Health and Safety Executive, Environment Agency, as well as invited groups. Their duty is to ‘plan, assess and advise’ on emergencies that threaten serious damage to human welfare (including property) and the environment (including plant life) [62]. The emphasis is on multi-agency collaboration.

Each Local Resilience Forum produces a public-facing Community Risk Register, which rates the relative likelihood, impact and risk of locally relevant hazards and threats over the next five years, and makes contingency plans to control them. The five-year span is short relative to the recurrence interval of severe wildfire incidents. Wildfire hazard is defined as ‘forest or moorland fire’ and local variants, and ‘severe wildfire’ applying to the rural–urban interface.^15^ Of the 41 Community Risk Registers in England and Wales for which data were publically available online in 2010, 31 included wildfire, with a modal rating of ‘medium risk’ (score 2 out of 5) [12]. Wildfires were therefore already widely recognized at regional level before the ‘severe’ category was added to the National Risk Register in 2013. This could be seen as a further example of the bottom-up evolution of wildfire risk awareness from regional to national, drawing on the experience of FRS representatives. In truth though, information flow is two-way; the National Risk Assessment considers significant emerging local risks identified by Local Resilience Forums in their Community Risk Registers, but in turn, Forums are required to consider hazards included in the National Risk Assessment. The process is facilitated by central representation on Forums, notably a DCLG resilience adviser, and by DCLG–Cabinet Office risk workshops every two years when a new National Risk Assessment and associated guidance is published.

### (c) Fire and Rescue Services response to wildfire

#### (i) Integrated Risk Management Plans

The introduction of the Fire and Rescue Service Act (2004) placed wider duties on Fire Authorities [63, ch. 21]. The most recent National Framework (2012) defines three clear priorities in England (table 2) [64]. It requires Fire Authorities to assess the full range of foreseeable FRS-related risks their areas face, to make provision for prevention and protection activities, and to respond to incidents appropriately.

Fire Authorities must develop an Integrated Risk Management Plan which considers any emergency, including wildfires, which could affect their community. This includes cross-border, multi-authority and national risks which, like wildfire, do not respect institutional boundaries. For example, the Sandhurst Fire of 2004 affected parts of both Surrey and Berkshire. Integrated Risk Management Plans have to demonstrate how prevention, protection and response activities might best be used to mitigate the impact of incidents on communities in a cost-effective way [64, p. 10]. Policy guidance on how this should be achieved for wildfire in Integrated Risk Management Plans was set out in 2008 [65].

Some FRS have embraced wildfire risk in their planning and prevention. Research undertaken in 2015 by the Chief Fire Officers Association at the request of the England and Wales Wildfire Forum, shows that wildfire risks defined in Community Risk Registers align reasonably well with Integrated Risk Management Plans. Of the now 54 Fire and Rescue Services surveyed across the UK, Channel Islands and Isle of Man, 67% said that wildfire was included in their Community Risk Register and also either in their Integrated Risk Management Plan, or their local or area service plans [12].^16^ The qualitative depth of planning and prevention for wildfire is, however, uncertain. Unlike other emergency services, the Fire Service does not have an external inspectorate. DCLG has instead largely devolved assurance in the running of Fire Authorities to the local level, backed by assessment by the National Audit Office [66,67], although their last inspection did not undertake a qualitative analysis of local FRS risk assessment planning. Whatever the inconsistencies, the Integrated Risk Management Planning process does represent the beginnings of a commitment to address the complex issue of wildfire in a significant way.

#### (ii) A collaborative approach by FRS

The participation of some FRS in local fire groups from the 1990s is evidence of growing awareness that they need to involve other stakeholders such as land owners and land managers to help reduce ignitions, and to assist with contingency planning and suppression, especially for rural FRS, which face the brunt of extreme wildfire events but have limited resources to cope. The 2004 Act, 2008 guidance and 2012 National Framework [63, pp. 5–65] crystallized this; all Fire Authorities were required to work with their communities and a wide range of partners in wildfire groups, to help identify and protect them from risks and prevent incidents from occurring. The concept of local collaborative cross-sector working with land managers to improve response to wildfire incidents therefore became enshrined in national policy.

At the national level, the Chief Fire Officers Association established a wildfire group in response to the extreme wildfire events of 2011 to share best practice among FRS and raise awareness of wildfire risk (table 1). Initiatives include: developing the Wildfire Operational Guidance for the Scottish Government [10]; introducing FireWise Communities [68] and working with international partners in Catalonia, France and elsewhere to develop wildfire training for UK firefighters [26, p. 92, 69]. The Association is also working alongside academic partners to refine the way wildfire is defined and evidenced using the Incident Recording System [12].

### (d) Forestry response to wildfire

The response of wider stakeholders to wildfire in the UK is illustrated by the Forestry Commission, which has an evident interest in avoiding loss of timber and amenity value. Woodland cover has significantly increased since 1905, with coniferous woodland accounting for just over half of the UK woodland area, although significantly higher in Scotland [70]. The Forestry Commission has led the way in raising awareness of wildfire as a land management issue (table 1). Their analysis of Incident Recording System data has already been discussed. Here, we present three further examples.

#### (i) UK Vegetation Fire Standard

The introduction of the Incident Recording System helped promote the emergence of standards for defining wildfire. Agreement on standards is crucial to systematic innovation across many sectors [71]. Forestry Commission England was asked by DCLG to help define relevant wildfire criteria for the Incident Recording System. The ‘UK Vegetation Fire Standard’ brought together datasets from wildfire incidents and prescribed burning practices [72]. Consultation was facilitated by both the Scottish and England and Wales Wildfire Forums. As a result, UK national reporting requirements were linked for the first time to United Nations and European requirements and should facilitate the inclusion of UK fire statistics into the European Forest Fire Information System [73]. These standards also provide a platform for integrated wildfire reporting across Forestry Commission England, Forestry Commission Scotland and Natural Resources Wales.

#### (ii) UK Forestry Standard

Forestry is a devolved issue, but standards and guidance are set at ‘UK’ level. A key example is the UK Forestry Standard, which helps to ensure that forestry is sustainable and meets international agreements and national legislation [74]. Legal requirements and good forestry practice are combined with guidelines for compliance from different elements of sustainable forestry management. The Standard requires planning for forest fires; for example, a contingency plan and building resilience through adaptation in age classes, species selection and stand structure. From this document, others cascade down, as discussed next.

#### (iii) Practice guidance: building wildfire resilience into forestry management planning

As a result of the 2011 Swinley Forest Fire, the Forestry Commission published practice guidance to help ensure both private and public Forestry Management Plans include mitigation and adaptation to wildfire incidents [75]. The aim was to move away from over-reliance on linear defences of fire breaks and fire plans to a more inclusive and integrated whole-site prevention approach.

The document anticipates the impacts of future climate change, especially in South East England. It covers wildfire behaviour, the need to plan for wildfire, and forest management plans for integrating wildfire resilience. It highlights forest management techniques to help prevent and improve response when wildfires do occur, including managing vegetation and fuels, creating fire breaks and fire belts and improving forest design; for instance, strategic placement of deciduous tree components to slow fire spread. Scope exists for building silvicultural resilience; deciduous trees are far more fire-resistant than young conifers. Forestry Commission plans for incident response include provision of circular fire access routes, water supplies and hard standings. The Practice Guide also provides tool kits for the planning process, including Wildfire Risk Assessment, Wildfire Management Zones, Wildfire Response Plans and Wildfire Management Plans.

This guidance has helped diffuse best practice on managing wildfire hazard across other sectors (table 1). For instance, applicants to DEFRA's^17^ Countryside Stewardship grant scheme on lowland heath must produce a Wildfire Risk Assessment and a Wildfire Response Plan agreed by the local FRS and must mitigate wildfire where it was identified as a risk in Environmental Statements in accordance with Forestry Commission practice [76]. It influenced the design of a major housing development adjacent to the Swinley Forest fire site and is recommended by Dorset FRS to help landowners reduce wildfire risk [77]. It was also used in the redesign of Dorset's Purbeck Forest.

#### (iv) Purbeck Forest (Dorset) case study

The Forestry Commission's scheme for Dorset's Purbeck Forest in Southern England shows how the potential impact of wildfire can be mitigated by collaborative planning. Partial deforestation of coniferous woodland to generate lowland heath was proposed—a shift to an open, more fire-prone and heavily used ecosystem. The Forestry Commission's 2010 ‘Open Habitats Policy’ required liaison with the local Fire Authority to agree appropriate mitigation and control measures. Fire would damage the ecosystem and pose a threat to the A35 Trunk Road and the Wytch Farm oil processing facility. An Environmental Impact Assessment screening exercise was undertaken and judged that an Environmental Statement [78] was required.

**Figure 2.**
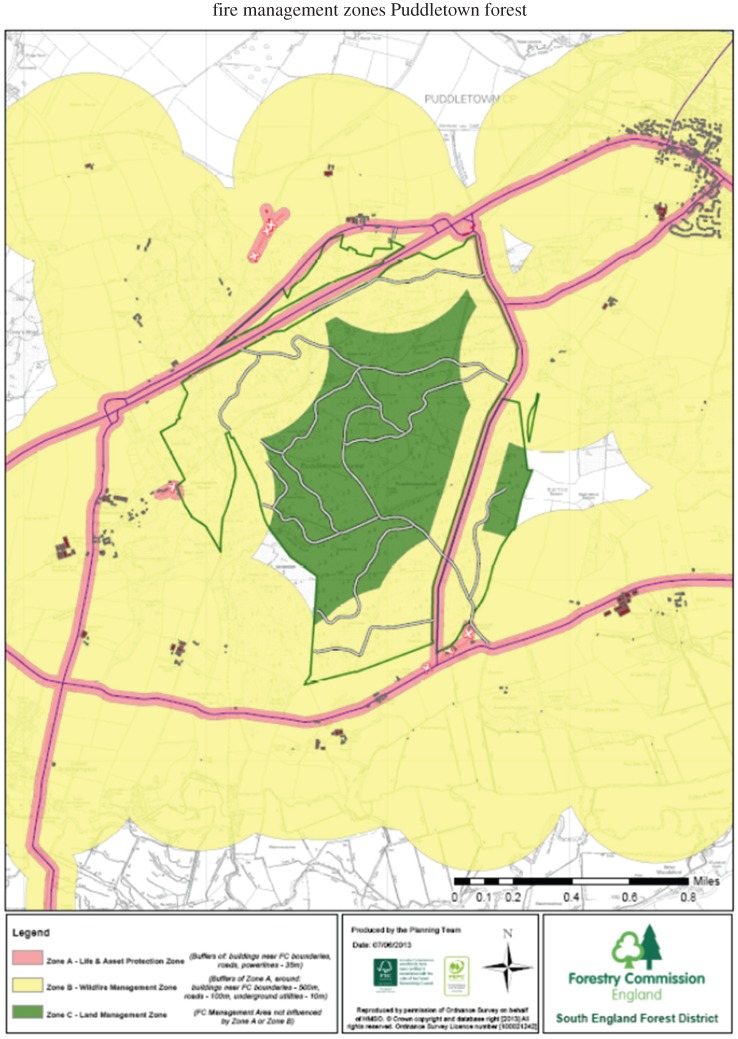
Puddletown wildfire management zones, Dorset (2013). Forestry Commission England.

The Environmental Statement showed that creation of open heathland would have a ‘moderate negative significance’ to wildfire resilience and proposed mitigation measures to reduce it to ‘minor negative significance’ [79]. The reduction was achieved using the Forestry Commission Practice Guidance to develop a Wildfire Risk Assessment, Wildfire Management Zones (figure 2) and Wildfire Response Plan, delivered in part by the landscape-scale Wild Purbeck Nature Improvement Area project in consultation with Dorset FRS and surrounding landowners [80]. In practice, this meant producing fire maps and an action plan, and local training in fighting wildfires.

### (e) Climate change as a catalyst for national cross-sector working

#### (i) UK Climate Change Risk Assessment

The danger of climate change has brought wildfire into sharp international focus [81]. The UK Climate Change Risk Assessment 2012 identified increased frequency of wildfire as one of seven key risks [7, p. 15]. It would have ‘medium negative consequence’ under their medium emissions scenario, anticipating warmer and drier conditions over the medium to long term (2050s and 2080s) [82]. However, this time scale is within the ‘short term’ of land management, especially for forestry. The risk overall could increase in the UK between 10% and 50% by the 2080s, with the greatest increase (over 40%) in South East England and concentrated into ‘wildfire seasons’. Wildfire was seen as a key cross-sector risk, being cited under biodiversity and ecosystem services [83] and at least three of the 11 other sector reports^18^, and therefore, one which requires integrated land use and emergency planning. Linkages with other natural hazards were also highlighted; for instance, tree pests and diseases may encourage fire spread [84].

Climate change will also bring increasing pressure on Fire and other Emergency Services. However, Government is not always consistent in its treatment of the new policy issue of wildfire. Sir Ken Knight's 2013 review of proposed Fire Service efficiencies and operations did not address the financial challenges of increasing frequency of wildfires, nor the impacts of a changing climate [85].

#### (ii) National Adaptation Programme

In response to the Climate Change Risk Assessment 2102, the National Adaptation Programme 2013 aimed to make the UK more resilient to a changing climate [86]. Knowledge and cross-sector structures within the England and Wales Forum were explicitly highlighted and used in the national plan. The Programme identified three vital roles of the Forum in preparing for the impact of climate change on wildfires: in issuing guidance to land managers to reduce the risk of wildfires occurring and effective response and recovery when they do; in building resilience by promoting more appropriate land use and habitat management; and in addressing gaps in knowledge and research.

The Climate Change Risk Assessment and National Adaptation Programme placed an action on FRS to review the risk of severe wildfire through the Integrated Risk Management Planning processes. In response, the Chief Fire Officers Association produced a report that acknowledged the present and future growing risks of large wildfire incidents, recommending that ideally the risk of severe fires should be considered, and where appropriate addressed, during the development of each relevant FRS Integrated Risk Management Plan [87]. Natural England also produced a report in which wildfire was considered a high-priority threat to landscapes and biodiversity as well as to public access and engagement [88]. It highlighted the increasing wildfire risk to heaths, stating that it would define how mitigation of wildfire risk would be encouraged in land management schemes for susceptible habitats. The climate change agenda has therefore been an important driver in raising awareness of wildfire as a cross-sector issue.

### (f) Development control planning and other under-represented sectors

Certain sectors and government departments are so far poorly engaged in wildfire risk management, including the Department of Energy and Climate Change, the insurance industry and, especially, development control planning (table 1). For development control planning, there is a risk that major residential developments will be situated next to high-risk wildfire sites in the rural–urban interface, posing a risk to public health and safety. For example, a pilot study of a sample area of 11 by 12 km around the area of the 2011 Swinley Forest fire showed there are 33 care homes for the elderly in the area, six of which are adjacent to fire-prone heathland [89].

There are examples of good practice using the planning system to help fund wildfire risk management, notably ‘Natura 2000 Heaths’ in Dorset [90], and as already cited, to mitigate the risk for a further housing and care home development adjacent to the Swinley Forest fire site using the Forestry Commission's Practice Guidance. However, planners' awareness of wildfire risk in the UK rural–urban interface remains low. This is because the planning process is reactive; it responds to severe events, and return periods for severe wildfire are typically longer than the political cycle. Policy instruments therefore do not specifically identify wildfire, and suitable tools to quantify wildfire risk are not yet widely available.

The National Planning Policy Framework for England and Wales was developed to simplify the numerous Planning Policy Statements and Guidance that have evolved over the past few decades, and to ensure a correct balance between sustainable social, economic and environmental development. It considers both climate change and natural hazards in planning policy and decisions (table 2) [91]. Policies 121 and 164, among others within the Framework, suggest that planning decisions should take account of vulnerability to ‘natural hazards’ in the generic sense. Policy 99 suggests planning authorities should anticipate the impact of climate change over the longer term. In all cases, natural hazards and climate change tacitly include wildfire, however, until it is overtly specified, wildfire will continue to be overlooked in most planning decisions. Further, no real traction is likely until tools are developed to quantify and map wildfire likelihood, impact and resulting risk in a similar way to flooding, for example, using wildfire threat analysis as adopted in New Zealand [92,93]. It took severe flooding before risk mapping tools were established. Regrettably, it will take more severe events like Swinley Forest to put wildfire firmly on Planning's radar.

Use of planning policy to help adaptation to wildfire is a common approach in North America and Australia. For example, the Australian States of Victoria (covering rural areas) and Tasmania (rural and urban) both use a partnership approach across the planning control/development and FRS [94,95]. Anticipation of wildfire is embedded into integrated land use planning, habitat management and building regulations. This joined-up approach builds resilience to all but the most extreme bushfire incidents.

With the pressure to build more houses, especially in South East England, greater emphasis in the planning system is placed on protecting surrounding priority species and habitats. Perversely, land management for purely conservation objectives can be inappropriate for wildfire mitigation and adaptation, creating greater risk to the public and the wider environment. Providing more green infrastructure also represents more fuel. Greater effort is needed to engage this sector. Otherwise, as one UK planning consultant put it ‘we will keep putting people closer to your risk—bringing risk closer to the fuel’.

## 4. Discussion and conclusion

### (a) Comparison with the USA

The response to wildfire in the UK remains varied, fragmented and incomplete at local level. National policy is related to goals such as disaster management and adaptation to climate change. Responsibility for the single problem of wildfire is fragmented across government departments. There is a need to overcome the challenges of complexity and fragmentation by introducing a clear policy, at least towards potential severe wildfires. In effect, policy needs to act as a selection mechanism to pick the best features of the community-based response at local level and to combine initiatives at the national level. To some extent a pragmatic solution is emerging as groups such as the England and Wales Wildfire Forum become *de facto* consultative panels for government. Of the 10 policy outcomes for managing wildfire risk in the rural?–urban interface presented at the Wildfire 2015 conference, local approaches for partnerships working and engagement as part of cross-sector working were voted as the top three by wildfire professionals, landowners, FRS delegates and researchers [96].

A different pattern of evolution in policy towards wildfire is apparent in the USA, where it has been described as a ‘process of adaptive governance mediated by institutions at multiple scales’ [35]. In the USA, however, a national policy framework already existed through Federal institutions such as the National Park Service [97]. Rather, local responses have recently emerged because of a need to align the scale of decision-making with direct experience of those who bear the consequences of those decisions. This adaptive response in the USA is an example of ‘scale matching’, the idea that environmental problems are best dealt with at the level where there is the best fit between social and ecological components of a system [98,99]. In the USA, there is a clear process of modifying existing institutions to respond better to local community needs.

In effect, the process of adaptive governance seen with wildfire in the USA has been reversed in the UK. Instead of governance spreading from central control towards local solutions, in the UK, the evolution of local solutions has prompted *ad hoc* coordination at local and national level. In turn, this has influenced the formal policy of government as it evolved through emergency planning and climate change legislation.

It is now argued that the USA needs to move towards a position where communities work hand in hand with planners, architects and land managers to coexist with wildfire [100]. These recommendations have strong echoes of the emergence of local wildfire groups in the UK during the 1990s and accord closely with the goals of the Forestry Commission in managing fire. However, as we have seen, the UK response still largely ignores the role of the UK planning system in anticipating wildfire problems and improving resilience.

### (b) Conclusion

The UK experiences wildfires annually, but the episodic frequency of severe incidents reduces awareness in wet years. Return periods for severe wildfire are typically longer than the political cycle and five-year time span considered for emergency planning. Historically limited fire statistics, especially for burnt area, hinder the ability to accurately evidence the issue and quantify risk. Development of agreed standards, pioneered by the Forestry Commission, allowed geo-referenced data on wildfires to be collected in the Incident Recording System for all vegetation fires across GB from 2009, although spatial accuracy and lack of reliable data on fire perimeters limit development of geographic information systems-based risk assessment tools at the local scale.

England does not yet have a specific national wildfire agency or strategy. The now 46 regional FRS have a statutory duty to extinguish wildfires alongside structural fires and other emergency rescue duties. The definition of wildfires is lenient, covering any uncontrolled vegetation fire where a decision or action regarding suppression is required. The Scottish government identifies the more significant incidents in the Incident Recording System using a definition based primarily on FRS resources and estimated burned area. For England, further work is needed to agree a hierarchy of vegetation fires, which allows for differences in local circumstances between FRS and also suits outcome scenarios used in Community Risk Registers.

The FRS-centred approach and resulting suppression paradigm runs the risk of more severe fires in the future, unless other methods of managing fuel and ignition sources are also implemented. This important international message is not widely realized in the UK. In this respect, recognition as hazard is a double-edged sword, denying beneficial effects of vegetation fire.

Successful management requires the adoption of a cross-sector approach at the national scale, not just as now for the emergency response phase of large incidents, but also at the prevention phase. As we have seen, this is beginning to be redressed with wildfire risk assessments now included as conditions of some agri-environment subsidies. Even now though, such a national cross-sector approach is challenging because of the fragmented responsibility for wildfire at different phases of the hazard chain. Policies which impact on wildfire have evolved separately in each sector and can result in unintended consequences.

In the absence of coordinated central policy guidance and spurred by individual champions, community-based solutions gradually emerged during the 1990s, long before formal awareness and government policy began to deal with the issue. Indeed, these grass-roots responses have diffused upward to facilitate later central government actions. This has happened at two levels; first, local and regional fire groups evolved in response to the crisis events of 1996, 2003, 2006 and 2011; and second, they were followed by national forums, aided by academia-led knowledge exchange initiatives. FRS facing problems of rural wildfire and limited financial resources were forced to work in partnership with land owners and managers, environmental groups, water authorities and other stakeholders, and to innovate at the local level. These local fire groups took ownership of the wildfire problem, collaborating to gradually develop knowledge and management strategies at scales matched to their own local social and ecological conditions. On a national scale, the England and Wales Wildfire Forum, Scottish Wildfire Forum and the Chief Fire Officers Association Wildfire Group are helping to spread good practice laterally and vertically, assisted by academic knowledge exchange initiatives. Knowledge of wildfire management has been co-produced by both scales of these self-assembling, informal partnerships, improving local emergency response on the ground and raising government awareness of wildfire. Both levels fit the evolutionary model in that participatory solutions gradually evolved in a cumulative way and vary between groups [8].

For national government, both the emergency planning and climate change agenda have been significant catalysts for wildfire awareness and the emergence of cross-sector working. Systematic national policy towards wildfire as hazard began to emerge in 2004, when Government initiated a programme of contingency planning against risks and natural hazards facing civil society. By 2010, most Community Risk Registers included ‘forest or moorland fire’. Crisis events have again been very significant; national awareness of wildfire was spurred by the 2011 fire season, and especially by the small but high-impact rural–urban interface fire at Swinley Forest. Risk assessments for the 2012 London Olympics also played a part. Severe wildfire was included in the National Risk Register for the first time in 2013. Wildfire is now included in the majority of Community Risk Registers and FRS Incident Risk Management Plans. The need for national cross-sector collaboration on wildfire was boosted by the Climate Change Risk Assessment in 2012.

Key stakeholders such as the Forestry Commission have pioneered good practice in adaptive land management to build fire resilience into UK forests by developing best practice guides and evidencing wildfire occurrence from national fire statistics. Their approach has begun to diffuse into local areas adjacent to woodlands, and into broader DEFRA policy for lowland and upland heath. The Dorset case study shows that potential impact of wildfire can be mitigated by an adaptive collaborative approach to landscape planning, and innovative, but rare and much needed engagement with development control planning.

In summary, policy and practice have responded slowly and fitfully to the complexity of the wildfire problem. Taken overall, wildfire policy exhibits an evolutionary process, where locally adapted participatory solutions have emerged at multiple levels and diffused in response to need, as much as to legislation. The current national policy paradigm is still one of fire suppression in keeping with FRS practice for structural fires. Recognition of severe wildfire as a national hazard has pushed it up the emergency planning agenda, but potentially undermines the longer-term benefits of vegetation fire and its role as a part of the socio-ecological system. We are still a long way from learning to live with fire, but the need for a risk management approach to wildfire, instead of zero tolerance to all vegetation fires is beginning to be recognized. Progress is being made towards a cross-sector approach that integrates fire and land management, especially at the prevention stage. The grass-roots evolution of participatory solutions has been a key enabling process. A coordinated and funded policy is now needed to identify best practice and promote understanding of the role of fire in UK socio-ecological systems.^19^

## Endnotes

1 The devolved administrations have often parallel but separate governance and legislation through the Scottish Parliament, the National Assembly for Wales of Scotland and the Northern Ireland Assembly. https://www.gov.uk/guidance/devolution-of-powers-to-scotland-wales-and-northern-ireland.

2 http://www.frsug.org/reports/Wildfire_IRS_2014.pdf (accessed 11 April 2016). Summary of Forestry Commission analysis of Incident Recording System for Wildfire evidence (2014) Forestry Commission, R.G., published on the Fire and Rescue Statistics User Group website. Figures are based on four financial years of Incident Recording System data for England, Scotland and Wales, 1 April 2009–31 March 2013. Northern Ireland fires were recorded separately, so are not included in this analysis.

3 ‘Urban’ comprises the following Incident Recording System property codes; 396 Domestic garden (vegetation not equipment), 397 Nurseries and market gardens, 450 Park and 453 Wasteland.

4 Royal Berkshire Fire and Rescue Service. http://www.rbfrs.co.uk/incidents.html (accessed 3 Nov 2015).

5 For example, Channel 4 News, UK fires blaze after hottest ever April, 4 May 2011. http://www.channel4.com/news/uk-fires-blaze-after-hottest-ever-april (accessed 12 Feb 2016).

6 http://www.frsug.org/reports/Wildfire_IRS_2014.pdf (accessed 11 April 2016).

7 http://www.frsug.org/reports/Wildfire_Statistics_2012.pdf. Forestry Commission, R.G., published on the Fire and Rescue Statistics User Group website. Figures are based on two financial years of Incident Recording System data for England, Scotland and Wales, 1 April 2009–31 March 2011. Northern Ireland fires are recorded separately, so were not included in this analysis. Based on total vehicle hours for all vegetation fire incidents at £300 per hour.

8 Up to financial year 2008/2009, fire statistics reports as compiled by the Department of Communities and Local Government (DCLG) covered the UK. Since 1 April 2009, they cover Great Britain only (https://www.gov.uk/government/collections/fire-statistics-great-britain) because Northern Ireland did not immediately adopt the Incident Recording System. In the Fire Damage Report (FDR) system, which previously operated UK-wide, full ‘FRD1 reports’ were only required for ‘primary’ vegetation fires; i.e. all fires in buildings, vehicles and outdoor structures, or any fire involving casualties, rescues or fires attended by five or more appliances. ‘Secondary’ fires (the majority of vegetation fires) were estimated from a sample. The Incident Recording System still flags primary fires, but reports all vegetation fires equally.

9 We are working with the Chief Fire Officers Association Wildfire Group and the England and Wales Wildfire Forum to refine this definition; e.g. [30]*.*

10 Also exemplified by DEFRA's former Best Practice Burning Group (now Uplands Management Group), a stakeholder consultation group of Natural England, other wildlife conservation groups and moorland managers, who co-produced the Heather and Grass Burning Code 2007, and now coalesce around the concept of *Sphagnum* regeneration and rewetting as a means to increase ecological resilience to wildfire risk.

11 http://www.northumberland.gov.uk/Fire/Wildfire.aspx?nccredirect=1 (accessed 22 Nov 2015).

12 www.fires-seminars.org.uk (accessed 22 Nov 2015).

13 www.kfwf.org.uk (accessed 22 Nov 2015).

14 Also known as Fire and Rescue Authorities. They oversee their regional FRS and are funded jointly by central Government and local rates; levied by Local Authorities on residents for provision of essential services, including FRS.

15 The outcome description (scenario) for ‘forest or moorland fire, HL33’ is across up to 50 ha, evacuation of up to 100 residential homes required, up to 5 fatalities and 20 casualties'. Some such as Northumbria, Gloucestershire, Merseyside and Hertfordshire included their own variants and codes for lower severity or spread into the rural–urban interface. The scenario for ‘severe wildfire, H58’ is ‘severe wildfire spreading over an area of 1500 ha at an urban–rural interface and lasting for 7–10 days. Fatality numbers are low (under 10) and casualty figures are between 50 and 100, primarily as a result of respiratory complaints and burns. Dense smoke would spread for a distance of a minimum of 3–5 miles resulting in short to medium term closures of motorways and/or major arterial roads and railway lines’. http://www.staffordshireprepared.gov.uk/Risk-Register/documents/151123-Staffordshire-Community-Risk-Register.pdf (accessed 12 Feb 2016).

16 Where a decision was deemed to be pending, it was counted is as a yes in this cross-tabulation. Replies were received from 50 of the current total of 54 FRS. Percentages are out of 54.

17 DEFRA Country Stewardship grant; Management of lowland heathland, https://www.gov.uk/countryside-stewardship-grants/management-of-lowland-heathland-lh1 (accessed 12 Feb 2016).

18 Forestry; Agriculture, Built Environment and Transport. http://randd.defra.gov.uk/Default.aspx?Menu=Menu&Module=More&Location=None&Completed=0&ProjectID=15747#RelatedDocuments (accessed 7 Nov 2015).

19 Research needs are reviewed in Roos *et al*. [101].

## References

[1] Cabinet Office. 2013 National Risk Register of Civil Emergencies, 2013 Edition. London: Cabinet Office.

[2] Oxborough N, Gazzard R 2011 Swinley forest fire. *Fire Risk Management*, 11–15 October.

[3] Radley J 1965 Significance of major moorland fires. Nature 215, 1254–1259. (10.1038/2051254a0)

[4] Tallis JH 1987 Fire and flood at Holme Moss: erosion processes in an upland blanket mire. J. Ecol. 75, 1099–1129. (10.2307/2260317)

[5] Anderson P 1986 Accidental moorland fires in the Peak District: a study of their incidence and ecological implications. Bakewell, UK: Peak District Moorland Restoration Project, Peak District National Park Authority.

[6] Albertson K, Aylen J, Cavan G, McMorrow J 2010 Climate change and the occurrence of moorland wildfires in the Peak District of the UK. Clim. Res. 45, 105–118. (10.3354/cr00926)

[7] Department for Environment, Food and Rural Affairs. 2012 UK climate change risk assessment: government report. London, UK: The Stationery Office. Presented to Parliament pursuant to section 56 of the Climate Change Act 2008. See https://www.gov.uk/government/uploads/system/uploads/attachment_data/file/69487/pb13698-climate-risk-assessment.pdf (accessed 7 Oct 2015).

[8] Metcalfe JS 1995 Technology systems and technology policy in an evolutionary framework. Camb. J. Econ. 19, 25–46.

[9] Nafilyan V. 2015 Office for National Statistics. *UK Natural Capital Land Cover in the UK*. http://www.ons.gov.uk/ons/dcp171766_398497.pdf (accessed 11 April 2016).

[10] The Scottish Government. 2013 Fire and rescue service wildfire operational guidance, 359 p. Edinburgh, UK: Fire and Rescue Services Division, Edinburgh http://www.scotland.gov.uk/Publications/2013/10/6118.

[11] Department for Communities and Local Government. 2012 Incident recording system—questions and lists. Version 1.6 – (XML Schemas v1-0p). London, UK: Communities and Local Government.

[12] McMorrow J, Gazzard R, Hedley P 2015 Wildfire risk in community risk registers, integrated risk management plans and incident recording system: how well do they match? In Wildfires 2015, Cambuslang, Glasgow, 10–11 November 2015 http://www.firescotland.gov.uk/your-safety/wildfires/2015-uk-wildfire-conference-presentations.aspx.

[13] House of Commons, 4 May 2011, Column 665, Dr Phillip Lee (Bracknell), The Prime Minister Hansard See http://www.publications.parliament.uk/pa/cm201011/cmhansrd/cm110504/debtext/110504-0001.htm#column_665.

[14] Holden J et al. 2007 Environmental change in moorland landscapes. Earth Sci. Rev. 82, 75–100. (10.1016/j.earscirev.2007.01.003)

[15] Rothwell JJ, Evans MG, Liddaman LC, Allott TEH 2007 The role of wildfire and gully erosion in particulate Pb export from contaminated peatland catchments in the southern Pennines, UK. Geomorphology 88, 276–284. (10.1016/j.geomorph.2006.11.011)

[16] Anderson P, Buckler M, Walker J 2009 Moorland restoration: potential and progress. In Drivers of environmental change in uplands (eds Bonn A, Hubacek K, Stewart J, Allott TEA), ch. 24, pp. 432–447. Abingdon, UK: Routledge.

[17] McMorrow J, Lindley S, Aylen J, Cavan G, Albertson K, Boys D 2009 Moorland wildfire risk, visitors and climate change: patterns, prevention and policy. In Drivers of environmental change in uplands (eds Bonn A, Hubacek K, Stewart J, Allott TEA), ch. 23, pp. 404–431. Abingdon, UK: Routledge.

[18] Davies GM et al. 2016 The role of fire in UK peatland and moorland management: the need for informed, unbiased debate. Phil. Trans. R. Soc. B 371, 20150342 (10.1098/rstb.2015.0342) 27216512 PMC4874417

[19] Hincks TK, Malmaud BD, Sparks RSJ, Wooster MJ, Lynham TJ 2013 Risk assessment and management of wildfires. In Risk and uncertainty assessment for natural hazards (eds Rougier J, Sparks S, Hill LJ). Cambridge, UK: Cambridge University Press.

[20] Davies GM, Gray A, Hamilton A, Legg CJ 2008 The future of fire management in the British uplands. Int. J. Biodivers. Sci. Manage. 4, 127–147. (10.3843/Biodiv.4.3:1)

[21] Stokes KE, Alchin AE, Bullock JM, Watkinson AR 2004 Population responses of Ulex shrubs to fire in a lowland heath community. J. Vegetation Sci. 15, 505–514. (10.1111/j.1654-1103.2004.tb02289.x)

[22] Hanley ME 2009 Thermal shock and germination in North-West European *Genisteae*: implications for heathland management and invasive weed control using fire. Appl. Vegetation Sci. 12, 385–390. (10.1111/j.1654-109X.2009.01038.x)

[23] Albertson K, Aylen J, Cavan G, McMorrow J 2009 Forecasting the outbreak of moorland wildfires in the English Peak District. J. Environ. Manage. 90, 2642–2651. (10.1016/j.jenvman.2009.02.011) 19321251

[24] De Jong MC, Wooster MJ, Kitchen K, Manley C, Gazzard R 2015 Calibration and evaluation of the Canadian Forest Fire Weather Index (FWI) System for improved wildland fire danger rating in the UK. Nat. Hazards Earth Syst. Sci. Discuss 3, 6997–7051. (10.5194/nhessd-3-6997-2015)

[25] Maltby E, Legg CJ, Proctor MCF 1990 The ecology of severe moorland fire on the North York Moors: effects of the 1976 fires, and subsequent surface and vegetation development. J. Ecol. 78, 490–518. (10.2307/2261126)

[26] Hedley P 2014 Addressing the UK's wildfire risk: a collaborative approach. *International Fire Fighter*. **41**, pp. 90–92.

[27] San-Miguel-Ayanz J, Moreno JM, Camia A 2013 Analysis of large fires in European Mediterranean landscapes: lessons learned and perspectives. For. Ecol. Manage. 294, 11–22. (10.1016/j.foreco.2012.10.050)

[28] McMorrow J 2011 Wildfire in the UK: status and key issues. In *Proc. of the second conf. on the human dimensions of wildland fire* (eds SM McCaffrey, CL Fisher), Gen. Tech. Rep. NRS-P-84. Newtown Square, PA: U.S. Department of Agriculture, Forest Service, Northern Research Station, pp 44–56. (http://www.nrs.fs.fed.us/pubs/gtr/gtr_nrs-p-84.pdf)

[29] Department for Communities and Local Government. 2015 Fire Statistics Great Britain: 2013 to 2014. https://www.gov.uk/government/statistics/fire-statistics-great-britain-2013-to-2014.

[30] Grundy S, McMorrow J 2015 Using the Incident Recording System to define wildfire in Great Britain. In Wildfires 2015, Cambuslang, Glasgow, 10–11 November 2015.

[31] Alcasena FJ, Salis M, Vega-García C 2015 A fire modeling approach to assess wildfire exposure of valued resources in central Navarra, Spain. Eur. J. For. Res. 135, 87–107. (10.1007/s10342-015-0919-6)

[32] Chuvieco E et al. 2010 Development of a framework for fire risk assessment using remote sensing and geographic information system technologies. Ecol. Model. 221, 46–58. (10.1016/j.ecolmodel.2008.11.017)

[33] McMorrow J 2013 MODIS-detected fire regime in Great Britain: potential and challenges of validating against national fire incident data. ‘Quantifying the Environmental Impact of Forest Fires’. In *Proc. EARSeL Forest Fire Special Interest Group workshop, Coombe Abbey, Warwickshire, 15–17 Oct 2013*, pp. 136–140. 10.5034/inquiryjrnl_50.01.02 23720879

[34] Millin-Chalabi G, McMorrow J, Agnew C 2014 Detecting a moorland wildfire scar in the Peak District, UK, using synthetic aperture radar from ERS-2 and Envisat ASAR. Int. J. Remote Sens. 35, 54–69. (10.1080/01431161.2013.860658)

[35] Abrams JB, Knapp M, Paveglio TB, Ellison A, Moseley C, Nielsen-Pincus M, Carroll MS 2015 Re-envisioning community-wildfire relations in the U.S. West as adaptive governance. Ecol. Soc. 20, 34 (10.5751/ES-07848-200334)

[36] Hedley P 2010 Working towards improving the UK's response to wildfire. *Fire Magazine*, May, pp. 34–35.

[37] Hedley P 2011 Towards an expanded lexicon. *Wildfire*, May/June, pp. 12–17.

[38] Carroll MS, Blatner KA, Cohn PJ, Todd M 2007 Managing fire danger in the forests of the US Inland Northwest: a classic ‘wicked problem’ in public land policy. J. For. 105, 239–244.

[39] McMorrow J et al. 2010 Fire Interdisciplinary Research on Ecosystem Services (FIRES) Policy Brief, 4p.

[40] Met Office Fire Severity Index http://www.metoffice.gov.uk/public/weather/fire-severity-index/#?tab=map.

[41] Badia A, Saurí D, Cerdan R, Llurdés J-C 2002 Causality and management of forest fires in Mediterranean environments: an example from Catalonia. Glob. Environ. Change B 4, 23–32. (10.1016/S1464-2867(02)00014-1)

[42] Miller JD, Safford HD, Crimmins M, Thode AE 2009 Quantitative evidence for increasing forest fire severity in the Sierra Nevada and Southern Cascade Mountains, California and Nevada, USA. Ecosystems 12, 16–32. (10.1007/s10021-008-9201-9)

[43] IUCN Peatland Programme UK http://www.iucn-uk-peatlandprogramme.org/publications/commission-inquiry/about-commission (accessed 22 Nov 2015)

[44] Douglas DJT, Buchanan GM, Thompson P, Amar A, Fielding DA, Redpath SM, Wilson JD 2015 Vegetation burning for game management in the UK uplands is increasing and overlaps spatially with soil carbon and protected areas. Biol. Conserv. 191, 243–250. (10.1016/j.biocon.2015.06.014)

[45] The Crop Residues (Burning) Regulations. 1993 http://www.legislation.gov.uk/uksi/1993/1366/contents/made.

[46] Aylen J 2009 Emergent innovation in public service—lessons from fighting wildfires. Public Money Manage. 29, 207–208. (10.1080/09540960903034943)

[47] Chilvers J, Evans J 2009 Understanding networks at the science–policy interface. Geoforum 40, 355–362. (10.1016/geoforum.2009.03.007)

[48] Garud R, Karnøe P 2003 Bricolage versus breakthrough: distributed and embedded agency in technology entrepreneurship. Res. Policy 32, 277–300. (10.1016/S0048-7333(02)00100-2)

[49] Witt U 2003 Economic policy making in evolutionary perspective. J. Evol. Econ. 13, 77–94. (10.1007/s00191-003-0148-x)

[50] Berthod O, Grothe-Hammer M, Sydow J 2015 Some characteristics of high-reliability networks. J. Contingencies Crisis Manage. 23, 24–28. (10.1111/1468-5973.12069)

[51] Rural Development Initiatives. 2010 Building our resilience to wildfire: preparation and prevention for 2012. Project Plan April 2010. Inverurie, UK: RDI for South East of England Regional Wildfire Group and Home Counties Operational Wildfire Group.

[52] Northumberland Fire Group See http://www.northumberland.gov.uk/Fire/Group.aspx (accessed 4 Nov 2015).

[53] North Pennines Wildfire Group See http://www.northpennines.org.uk/Pages/WildfireGroups.aspx (accessed 4 Nov 2015).

[54] Bulkeley H 2000 Discourse coalitions and the Australian climate change policy network. Environ. Plann. C 18, 727–748. (10.1068/c9905j)

[55] Hajer MA 1995 The politics of environmental discourse: ecological modernization and the policy process. Oxford, UK: Clarendon Press.

[56] Amin A, Roberts J (eds). 2008 Community, economic creativity and organization. Oxford, UK: Oxford University Press.

[57] Legg C, Davies GM FireBeaters Phase II Final Report to Scottish Natural Heritage. Project 23183, University of Edinburgh, School of Geosciences. https://www.era.lib.ed.ac.uk/bitstream/handle/1842/7640/firebeaters_II_final_report.PDF?sequence=1&isAllowed=y (accessed 22 Nov 2015).

[58] Dold J et al. 2010 Report on field experiments in Northumberland, March 2010: a multidiscipinary approach to assess fire behaviour and effects in a humid temperate climate. In *Proc. VI Int. Conf. on Forest Fire Research, Coimbra, Portugal, 15–18 Nov 2010*.

[59] HM Government. 2004 Civil Contingencies Act 2004, ch. 36 London, UK: HMSO http://www.legislation.gov.uk/ukpga/2004/36/contents.

[60] Cabinet Office. 2015 National Risk Register of Civil Emergencies, 2015 edn. London, UK: Cabinet Office.

[61] Cabinet Office. 2011 Keeping the country running: natural hazards and infrastructure. A guide to improving the resilience of critical infrastructure and essential services. London, UK: Cabinet Office Civil Contingencies Unit.

[62] Cabinet Office. 2013 Expectations and indicators of good practice set for category 1 and 2 responders: The Civil Contingencies Act (2004), its associated Regulations (2005) and guidance, the National Resilience Capabilities Programme, and emergency response and recovery. London, UK: Cabinet Office Civil Contingencies Secretariat.

[63] HM Government. 2004 Fire and Rescue Service Act (2004). London, UK: HMSO.

[64] Department for Communities and Local Government. 2012 Fire and rescue national framework for England. London: DCLG.

[65] Department for Communities and Local Government. 2008 Guidance integrated risk management planning guidance for fire and rescue authorities: wildfire. London, UK: Communities and Local Government.

[66] Department for Communities and Local Government. 2009 *op. cit*, p. 23.

[67] National Audit Office Report. 2015 Financial sustainability of fire and rescue services. London, UK: DCLG https://www.nao.org.uk/wp-content/uploads/2015/11/Financial-sustainability-of-fire-and-rescue-services.pdf (accessed 22 Nov 2015).

[68] National Fire Protection Association. 2014 Firewise Communities Moves Forward in the UK. See http://wildfire.blog.nfpa.org/2014/09/firewise-communites-moves-forward-in-the-uk.html (accessed 22 Nov 2015).

[69] Hedley P 2015 CFOA Wildfire Group learns from southern France study visit. *Fire Times*, Aug/Sept, pp. 52–53.

[70] Forestry Commission. 2015 Forestry Statistics 2015: a compendium of statistics about woodland, forestry and primary wood processing in the United Kingdom. Edinburgh, UK: Forestry Commission.

[71] Blind K 2004 The economics of standards; theory, evidence, policy. Cheltenham, UK: Edward Elgar.

[72] Gazzard RJ 2009 United Kingdom vegetation fire standard: data fields and terminology for wildfire incidents and prescribed burning operations within Great Britain and Northern Ireland. Farnham, UK: Forestry Commission http://www.forestry.gov.uk/pdf/UKVFS_August_2009.pdf/$FILE/UKVFS_August_2009.pdf (accessed 14 Oct 2015).

[73] http://forest.jrc.ec.europa.eu/effis/applications/fire-history/.

[74] Forestry Commission. 2011 The UK Forestry Standard: the governments’ approach to sustainable forestry, 3rd edn Edinburgh, UK: Forestry Commission.

[75] Forestry Commission. 2014 Building wildfire resilience into forest management planning: Forestry Commission Practice Guide. Edinburgh, UK: Forestry Commission.

[76] Natural England, DEFRA, Forestry Commission. 2015 Countryside stewardship grant management of lowland heathland (LH1). https://www.gov.uk/countryside-stewardship-grants/management-of-lowland-heathland-lh1 (accessed 22 Nov 2015).

[77] Dorset Fire and Rescue Service. Undated. Wildfire safety http://www.dorsetfire.gov.uk/safety/fire-road-safety/wildfire-safety/ (accessed 22 Nov 2015).

[78] Forestry Commission. 2009 Environmental assessment of forestry projects. Edinburgh, UK: Forestry Commission, Grants and Licences, FCCS053.

[79] Land Use Consultants. 2013 Purbeck forest design plan: heathland restoration proposals (2012–2026) Environmental Statement 2013. Bristol, UK: Land Use Consultants on behalf of the Forestry Commission.

[80] Wild Purbeck Nature Improvement Area. 2015 Wild Purbeck 2012–2015: final report 2015. Dorchester, UK: Dorset Area of Outstanding Natural Beauty.

[81] Krawchuk MA, Moritz MA, Parisien M-A, Van Dorn J, Hayhoe K 2009 Global pyrogeography: the current and future distribution of wildfire. PLoS ONE 4, e5102 (10.1371/journal.pone.0005102) 19352494 PMC2662419

[82] Department for Environment, Food and Rural Affairs. 2012 The UK Climate Change Risk Assessment 2012 evidence report. London, UK: DEFRA, section 3.2.7, p50; figures ES1 p ix and 8.1 p. 253 http://randd.defra.gov.uk/Document.aspx?Document=TheUKCCRA2012EvidenceReport.pdf (accessed 7 Nov 2015)

[83] Ridder B et al. 2012 Climate Change Risk Assessment for the biodiversity and ecosystem services sector. London, UK: DEFRA Project code GA0204, section 4.12. p. 113 http://randd.defra.gov.uk/Document.aspx?Document=CCRASummaryBiodiversityandEcosystemServices.pdf

[84] Moffat A, Morison J, Nicoll B, Bain V 2012 Climate Change Risk Assessment for the forestry sector. London, UK: DEFRA Section 4.5, p. 44 http://randd.defra.gov.uk/Document.aspx?Document=CCRAfortheForestrySector.pdf

[85] Department for Communities and Local Government. 2013 Facing the future: findings from the review of efficiencies and operations in fire and rescue authorities in England (The Knight report). London, UK: HMSO.

[86] HM Government. 2013 The National Adaptation Programme—making the country resilient to a changing climate. London, UK: The Stationery Office, p6, p56.

[87] Chief Fire Officers Association. 2012 Climate change adaptation report. Tamworth, UK: CFOA.

[88] Natural England. 2012 Natural England’*s climate change risk assessment and adaptation plan* York, UK: Natural England, General Publication Number NE318 http://publications.naturalengland.org.uk/publication/216300 (accessed 7 Nov 2015).

[89] Kazmierczak A, McMorrow J, Aylen J 2014 Developing a risk assessment approach for forest fire at the rural-urban interface: potential of the wildfire threat analysis framework. Final report. Manchester, UK: University of Manchester, School of Environment, Education and Development, September 2014, 78 p.

[90] Kazmierczak A, Carter J 2010 Dorset: financial contributions of planning applications to prevent heathland fires. In Adaptation to climate change using green and blue infrastructure: a database of case studies, pp. 69–78. Manchester, UK: University of Manchester http://www.grabs-eu.org/membersArea/files/Database_Final_no_hyperlinks.pdf.

[91] Department for Communities and Local Government. 2012 National Planning Policy Framework, 65 p. London, UK: DCLG https://www.gov.uk/government/publications/national-planning-policy-framework--2 (accessed 22 Nov 2015).

[92] Majorhazi K 2011 Wildfire threat analysis workbook, v3.1, 96pp. http://www.nrfa.org.nz/Operational%20documents/WTA_Wookbook.pdf (accessed 22 Nov 2015).

[93] Moffat AJ, Peace HG 2013 *Harmonising approaches to evaluation of forest fire risk:* NZ TRANZFOR Visit: Final Report. Forest Research, Farnham. http://kfwf.org.uk/_assets/documents/Moffat_and_Pearce_2013_Harmonising_approaches_to_evaluation_of_forest_fire_risk.pdf (accessed 21 Nov 2015).

[94] State of Victoria, Department of Transport, Planning and Local Infrastructure. 2015 Building in bushfire prone areas. http://www.dtpli.vic.gov.au/planning/planning-and-building-for-bushfire-protection/building-in-bushfire-prone-areas (accessed 22 Nov 2015).

[95] Bushfire Planning Group. 2005 Living with fire in Tasmania. *Guidelines for development in bushfire prone areas of Tasmania*, 24pp Hobart, Australia: Tasmania Fire Service http://www.fire.tas.gov.au/publications/Bush_Guide.pdf (accessed 22 Nov 2015).

[96] Gazzard R, McMorrow J, Stacey R 2015 Stakeholder priorities for managing wildfire risk in the Rural-Urban Interface (RUI): widening the consultation. In *Workshop at Wildfires 2015, Scottish Fire and Rescue Service Headquarters, Cambuslang, Glasgow, 10–11 Nov 2015* http://www.firescotland.gov.uk/your-safety/wildfires/2015-uk-wildfire-conference-presentations.aspx.

[97] Rothman HK 2007 Blazing heritage: a history of wildland fire in the national parks. Oxford, UK: Oxford University Press.

[98] Ager AA, Kline JD, Fischer AP 2015 Coupling the biophysical and social dimensions of wildfire risk to improve wildfire mitigation planning. Risk Anal. 35, 1393–1406. (10.1111/risa.12373/) 25968881

[99] Chaffin BC, Gosnell H, Cosens BA 2014 A decade of adaptive governance scholarship: synthesis and future directions. Ecol. Soc. 19, 56 (10.5751/ES-06824-190356)

[100] Smith AMS et al. 2016 The science of firescapes: achieving fire-resilient communities. Bioscience 66, 130–146. (10.1093/biosci/biv182) 29593361 PMC5865631

[101] Roos CI et al. 2016 Living on a flammable planet: interdisciplinary, cross-scalar and varied cultural lessons, prospects and challenges. Phil. Trans. R. Soc. B 371, 20150469 (10.1098/rstb.2015.0469) 27216517 PMC4874422

